# HTRA1 synergizes with oxidized phospholipids in promoting inflammation and macrophage infiltration essential for ocular VEGF expression

**DOI:** 10.1371/journal.pone.0216808

**Published:** 2019-05-17

**Authors:** Zhigang Lu, Victor Lin, Adam May, Briana Che, Xu Xiao, Daniel H. Shaw, Fei Su, Zhihao Wang, Hongjun Du, Peter X. Shaw

**Affiliations:** 1 Department of Ophthalmology, Altman Clinical and Translational Research Institute, University of California San Diego, La Jolla, CA, United States of America; 2 Department of Emergency Medicine, Sichuan Province People’s Hospital, Chengdu, Sichuan, China; 3 Westview High School, San Diego, CA, United States of America; 4 Department of Ophthalmology, Xijing Hospital, Xi’an, China; University of Florida, UNITED STATES

## Abstract

Understanding oxidative stress and HTRA1 locus in abnormal angiogenesis resulting in wet AMD pathology is an important step in developing a novel therapeutic approach. Using subretinal injection of oxLDL into C57BL/6 mice, we observed a lesion resembling the features of choroidal neovascularization (CNV), including macrophage infiltration, increased VEGF expression, and neovascularization. However, incubating ARPE-19 cells with oxLDL–a carrier of oxidized phospholipids–resulted in increased expression of inflammatory cytokines and chemoattractant proteins that recruited monocytes, but no substantial increase in expression of VEGF. Furthermore, incubation of ARPE-19 with oxLDL induced higher expression of HTRA1, which we showed to synergize with oxLDL in elevating the expression of inflammatory cytokines and chemoattractant factors. To investigate the role of macrophage infiltration on these expression changes, we treated cultured J774 macrophages with oxLDL and applied the conditioned medium onto ARPE-19 cells. This treatment was found to greatly enhance the expression of VEGF in ARPE-19, indicating the necessity of macrophage secretory products to induce increased expression of VEGF in retinal pigment epithelium. Gene expression analysis revealed that oxLDL induced the expression of Wnt3A in macrophages, a key activator of canonical Wnt signaling pathways. In addition, western blot analysis showed that the macrophage conditioned media further enhanced the reduction of phosphorylated β-catenin induced by oxLDL. Lastly, we investigated HTRA1 as a potential target for AMD therapeutics. We demonstrated the ability of anti-HTRA1 antibody *in vitro* to neutralize the protease activity of HTRA1 and reduce the inflammatory and angiogenic response to oxidative stress. Finally, we validated the neutralizing effect of anti-HTRA1 antibody *in vivo* by evaluating lesion size and protein expression in a laser-photocoagulation murine model of CNV. We found that the combination of oxLDL and HTRA1 enhanced CNV size, which was reversed by the addition of anti-HTRA1 antibody. This study not only provides preliminary evidence that HTRA1 may be a viable target for AMD therapeutics but also elucidates the biochemical mechanisms by which this therapeutic effect may be mediated.

## Introduction

Age-related macular degeneration (AMD) is the leading cause of blindness among elderly European-descended populations.[1] In early 2000, 1.75 million individuals in the United States and 3.35 million individuals in Western Europe were estimated to be affected by AMD, with these numbers projected to increase as populations age.[1] AMD is a late-stage form of age-related maculopathy (ARM), which is characterized by either the presence of subretinal yellow deposits with indistinct borders known as “soft” drusen or regions of hyper-/hypo-pigmentation of the retinal pigment epithelium (RPE) associated with drusen.[2] In its advanced stage, AMD can be classified into two major types: dry AMD and wet AMD. Dry AMD–also known as geographic atrophy–is characterized by regions of hypopigmentation or depigmentation of the RPE at least 175 um in diameter.[2] Wet AMD–also known as neovascular, exudative, or disciform AMD–is characterized by choroidal neovascularization within the subretinal space, resulting in the appearance of subretinal hemorrhages, neovascular membranes, scar tissue, fibrin-like deposits, hard exudates, or RPE detachment within the macula. While the clinical features of AMD have been studied for over a century, the pathogenesis of both disease types is still poorly understood. The purpose of this study is to investigate the underlying biochemical and cellular mechanisms that give rise to wet AMD and to propose a novel therapeutic approach that could be used to treat patients with wet AMD.

Numerous studies have identified both environmental and genetic risk factors for AMD. Many of the environmental risk factors for AMD–including smoking [3] and sunlight exposure [4]–result in increased levels of oxidative stress. Compared to other tissues, the retina is particularly predisposed to oxidative stress. First, the retina has a high metabolic demand from oxygen consumption. Second, the retina faces long-term exposure to sunlight, which can catalyze photooxidation reactions.[5, 6] Third, photoreceptor outer segments are highly enriched in unsaturated phospholipids that are prone to oxidation by reactive oxygen species or photooxidation by incident light.[7–9] The production of these oxidized phospholipids (oxPLs) results in the generation of chemical moieties that resemble epitopes on bacterial membranes–so-called “neo-epitopes”–and are recognized as foreign by the body’s innate immune system.[10, 11] This initiates activation of the complement cascade and release of cytokines and chemokines that recruit immune cells to the retina.[12–14] In patients with wet AMD, vascular endothelial growth factor (VEGF) is significantly upregulated [15, 16] and has been successfully targeted by antibody therapeutics in order to slow the progression of the disease.[17]

Besides these environmental insults, multiple gene variants have been associated with AMD.[18] One of the genes most strongly associated with AMD is that encoding high temperature requirement A1 (HTRA1).[19–21] HTRA1 is a serum protease that may modulate AMD pathogenesis through multiple pathways. Multiple substrates of HTRA1 have been identified, including multiple extracellular matrix (ECM) proteins and TGF-gamma family members.[19, 22] Previous studies have identified a disease haplotype–an SNP within the HTRA1 promoter on chromosome 10q26 –that influences risk for AMD.[20, 21, 23] While the association of HTRA1 variants with AMD has been well-established, the role of HTRA1 in AMD pathogenesis is poorly understood. A study by Hara *et al*. has found that HTRA1 variants with diminished proteolytic activity were associated with dysregulated vascular growth in the brain, causing a non-hypertensive cerebral small-vessel anteriopathy.[24] In our previous study, we observed significant downregulation of VEGF in both neuroretina and RPE and decreased retinal vasculature in HTRA1 knockout (htra1^-/-^) mice compared to wild-type (wt) mice.[25] Conversely, Jones *et al*. reported significant upregulation of VEGF and observed polypoidal choroidal vasculopathy (PCV), a variant of choroidal neovascularization, in transgenic mice overexpressing human HTRA1 (hHTRA1^+^).[26] Taken together, these three studies provide strong evidence that HTRA1 activity plays a pivotal role in choroidal neovascularization for patients with wet AMD.

In addition to promoting choroidal neovascularization, HTRA1 expression seems to be coupled to oxidative stress in the retina. Smoking, a well-known source of oxidative stress, has been shown to increase the odds ratio for developing choroidal neovascularization by 3.8, regardless of genotype.[27] However, when combined with the HTRA1 risk genotype, the odds ratio for developing choroidal neovascularization increases almost 50-fold.[27] Furthermore, in a study of rhesus macaques that develop drusen-associated age-related maculopathies, a variation in the promoter for the HTRA1 locus was shown to be significantly associated with drusen formation.[28] Proteomic analysis of drusen in humans has shown these lipid deposits to be enriched in oxidized proteins and lipids and are well-characterized participants in disease progression.[29] These studies implicate HTRA1 in the capacity of the retina to respond to oxidative stress.

This preliminary model of AMD pathogenesis–a dysregulated immune response to oxidative stress–is supported by studies in both humans and animals. However, we have recently observed that culturing ARPE19 cells with oxidized low density lipoprotein (oxLDL) stimulates the expression of inflammatory cytokines and their receptors, including IL-6, IL-8, CCR2, and MCP1. We have also noticed that, in contrast to subretinal injection of oxLDL in C57/BL6 mice that results in a CNV like lesion with upregulation of VEGF, the direct incubation of ARPE19 cells with oxLDL did *not* significantly upregulated VEGF expression. Our observation that there was a peculiar difference between in vitro and in vivo responses to oxidative stress prompt us to investigate the influence of oxidative stress and HTRA1 genetic variant with a novel perspective. We hypothesized that *in vivo* upregulation of VEGF is mediated with the infiltrating macrophages, which secret molecular signaling to activate Wnt signaling pathway, but are not incorporated into ARPE19 monoculture systems. In order to test this hypothesis, we cultured J774 macrophages with oxLDL and transferred the conditioned medium to the ARPE19 culture. Following incubation of the ARPE19 culture with the conditioned medium, the cells were collected and expression levels were measured via qPCR and Western blot.

In this report, we also seek to inhibit HTRA1 activity with antibodies to interrupt the association to oxPLs and/or RPE. We added the anti-HTRA1 antibody to the stimulation medium and then evaluate the expression of inflammatory factors and VEGF. The antibody targeting HTRA1 can greatly neutralize the HTRA1’s enzymatic activity and the ability to stimulate inflammation indicating that HTRA1 can be the potential target for treating wet AMD. As a proof of concept, we performed laser-induced CNV to evaluate the impact of anti-HTRA1 antibody on the CNV sizes. We found the addition of anti-HTRA1 antibody significantly reduced laser-CNV.

## Materials and methods

### Animals

All animals were treated in accordance with the National Institutes of Health Guide for the Care and Use of Laboratory Animals and the Association for Research in Vision and Ophthalmology (ARVO) statement for the Use of Animals in Ophthalmic and Vision Research. The animal use protocol is approved by the Institutional Animal and Care Use Committee (IACUC) of UC San Diego. Anesthetized mice were humanely euthanized by cervical dislocation followed by a bilateral pneumothorax, as detailed in our IACUC-approved protocol (s17017).

### Tissue culture

ARPE-19, a spontaneously arising RPE cell line that expresses the RPE-specific markers CRALBP and RPE-65, was obtained from the American Type Culture Collection (CRL-2302; ATCC, Manassas, VA). The macrophage cell line J774 (catalog # 67-TIB; ATCC) is a well-established model systems in cell biology and immunology.[30] ARPE-19 or J774 cells were cultured in Dulbecco's modified Eagle's medium-F12 (DMEM/F12) (ThermoFisher Scientific, Waltham, MA), supplemented with 10% Foundation B fetal bovine serum (FBS) (Catalog # 900, Gemini Bio-products, West Sacramento, CA) and 1% Penicillin-Streptomycin (Catalog #: PS-20, Omega scientific, Tarzana, CA) at 37°C in a humidified chamber with 5% CO_2_.

### Recombinant protein production and quantification

The pGEX plasmids harboring HTRA1 genes including the WT (containing a.a 114-C terminal due to the *E*. *coli* toxicity of the N-terminal), s328a (a.a. 328 serine is replaced by arginine to abolish protease activity), and 302x (truncation from a.a. 302 to removes PDZ domain) were kindly provided by Dr. Onodera.[24] The recombinant HTRA1 proteins were produced by IPTG induction of BL21 *E*. *coli* cells that were transformed with above plasmid. Twelve hours post IPTG induction, the cells were centrifugally collected then lysed by sonication. A glutathione Sepharose (Biovision 6555–10, San Francisco, CA) based column chromatography was performed on the lysate to purify and extract the recombinant HTRA1 protein which consists GST tag. The recombinant proteins were quantified using Micro BCA Protein Assay Kit (ThermoFisher Scientific, Waltham, MA #23235). The molecular weight was also confirmed with SDS-gel electrophoresis and Western blotting.

### Preparation of native and oxidized phospholipids

Since phospholipids have a very low aqueous solubility, we used LDL as a carrier for native and oxidized phospholipids in our study. In this routine method, LDL was prepared from plasma of normolipidemic donors by sequential ultracentrifugation.[31] OxLDL was generated by incubating LDL (1 mg/mL) with an oxidation agent (10 μM CuSO_4_) for 18 hours at 37°C where phospholipids on the surface of LDL are oxidized into oxPLs.[32] Native (un-oxidized) phospholipids on native-LDL was used as a control.

### Stimulation of RPE cells

Before treatment, the ARPE-19 or J774 cells were grown in DMEM/F12 (1:1) plus 10% FCS and 1% Penicillin-Streptomycin until uniformly reached 80% confluency. After serum starvation in DMEM/F12 (1:1) plus 0.1% BSA for 24 h, cells were stimulated with oxLDL or native-LDL as control at 50μg/ml for 12 hours, 24 hours and 48 hours. The cells were then washed once with cold PBS and collected with robber policemen for mRNA or protein analysis.

### Quantitative PCR

Total RNA was extracted from the cell lysates using Direct-zol RNA MiniPrep (ZYMO research, Irvine, CA). The RNA extract was converted to cDNA using High-Capacity cDNA Reverse Transcription Kit from Applied Biosystems. The converted cDNA was used as template for qPCR experiments using Power SYBR Green qPCR Master Mix from Applied Biosystems (Foster City, CA) with primer sets for HTRA1, VEGF and inflammatory cytokines/chemokines known to enhance CNV, including IL1, IL6, IL8, CCR2 (Table 1). Relative mRNA levels were normalized with GAPDH. The relative gene expression were quantified from qPCR results using the 2^(−ΔΔCT) method with the following formula:[33]

$$\begin{array}{l} \Delta CT=CT(a\;target\;gene)-CT(a\;reference\;gene).\\ \Delta \Delta CT=\Delta CT(a\;target\;sample)-\Delta CT(a\;reference\;sample)\\ Fold\;Change=2^{-\Delta \Delta CT} \end{array}$$

**Table 1:** qPCR primer sequences.

**CD36**	FW 5’-CAGAGGCTGACAACTTCACAG
	BW 5’-AGGGTACGGAACCAAACTCAA
**MCP-1**	FW 5’-TCTGTGCCTGCTGCTCATAG
	BW 5’-AGATCTCCTTGGCCACAATG
**CCR2**	FW 5’-AGAGGCATAGGGCAGTGAGA
	BW 5’-GCAGTGAGTCATCCCAAGAG
**VEGF**	FW 5’-TCCCGGTATAAGTCCTGGAG
	BW 5’-ACAAATGCTTTCTCCGCTCT
**IL6**	FW 5’-AAATTCGGTACATCCTCGACGG
	BW 5’-GGAAGGTTCAGGTTGTTTTCTGC
**IL8**	FW 5’-TCTGCAGCTCTGTGTGAAGG
	BW 5’-AATTTCTGTGTTGGCGCAGT
**Htra1**	FW 5’- CAGCCACTATGTATCACACG
	BW 5’- ACTGCAACCTACATATCCCG
**WNT3a**	FW 5’-GTTCGGGAGGTTTGGG
	BW 5’-CCAGGAAAGCGGACC
**Beta-Catenin**	FW 5’-ACAAACTGTTTTGAAAATCCA
	BW 5’-CGAGTCATTGCATACTGTCC
**GAPDH**	FW 5’-GAGTCAACGGATTTGGTCGT
	BW 5’-GACAAGCTTCCCGTTCTCAG

### Western blotting

The ARPE-19 or J774 cells were be lysed to examine the target genes using SDS-PAGE and Western blot analysis.[25] Cellular lysate was prepared using RIPA lysis buffer with phosphatase and protease inhibitors (Cell Signaling Tech. Danvers, MA) and quantified for total protein by BCA assay with a standard curve generated using a BSA standard (ThermoFisher Scientific). Same amount of protein samples was resolved by SDS-PAGE then transferred onto PVDF membrane, and probed using antibodies against either β-Catenin or phosphorylated β-Catenin that detected the ser37 phosphorylation site (Cell Signaling Tech.). Anti-GAPDH was used for an internal control. The HRP conjugated anti-Rabbit IgG was used as secondary antibody followed by SuperSignal West Pico PLUS Chemiluminescent Substrate (ThermoFisher Scientific). The ImageQuant LAS 4000 (GE healthcare, Chicago, IL) was used for signal quantification, and ANOVA with post-hoc t-tests was used to evaluate statistical significance.

### Intravitreal injection of oxLDL and eye tissue lysate preparation

Wild-type C57/BL6 mice were used to investigate the expression of VEGF in the eye under the stimulation of oxLDL or HTRA1 protein through intravitreal injection. Prior to injection, mice were anesthetized with intraperitoneal injection of a mixture of ketamine and xylazine. Intravitreal injections of 2μl different compound were performed immediately. The final concentrations of compounds for in vitreous injection of Na-LDL and oxLDL was 50 μg/ml; HTRA1 was 100 ng/ml; and anti-HTRA1 Ab was 1 μg/ml. One week post injection, the animals were sacrificed with CO_2_ and the eyes were removed. After washing with cold PBS buffer, the eyes were dissected by removing extra tissues, optic nerves and lens. The eyes were then minced and lysed with RIPA buffer (200 μl for each eye) followed by sonication. After spinning the lysate for 10 minutes (13,000 rpm at 4°C), the supernatant was collected for ELISA assay.

### Sandwich ELISA to detect VEGF

ImmunoGrade 96-well immunoassay microplate (BRANDplates, Sigma-Aldrich, St. Louis, MO) were coated with 2 μg/ml Rabbit Polyclonal VEGF Antibody (Novus Biologicals NB100-2381SS) in carbonate coating buffer (0.15 M sodium carbonate, 0.35 M sodium bicarbonate, pH 9.6) overnight at 4°C. After aspirating the coating solution, the plate was blocked in blocking buffer (PBS with 1% BSA, 0.1% Tween 20) at 37°C for 2 hours followed by three washes with washing buffer (PBS, 0.05% Tween-20). The samples were prepared in dilution buffer (PBS, 0.05% Tween-20, 0.1% BSA). The diluted samples of 10 ug/ml were then added to the wells and incubated in 37°C for 90 minutes. After three washes, the primary antibody: Mouse anti-VEGF antibody (Santa Cruz Biotechnology SC-53462) of 2μg/ml in dilution buffer was added and incubated at room temperature for 2 hours. After three washes, horseradish peroxidase-conjugated Goat anti-mouse IgG (Bio-Rad #1806516) of 10μg/ml in dilution buffer was added and incubated at room temperature for 90 minutes. After four washes, the plates were incubated with SuperSignal ELISA Pico Substrate at room temperature and the luminescence was measured in relative light units (RLU) using Filter-Max F3 plate reader (Molecular Devices, San Jose, CA).

### Subretinal injection of oxLDL

Wild-type C57/BL6 mice were used to investigate the effect of *local* oxidative stress induced by oxPLs on the induction of AMD-like features in the retina, and the role of HTRA1 in this process. We have previously published that subretinal injection of oxLDL causes CNV-like features in mice.[10] Four-week old mice from each background were subjected to injection of oxLDL in one eye, while the contralateral eye received native-LDL as a control. Prior to injection, mice were anesthetized with intraperitoneal injection of a mixture of ketamine and xylazine. The pupil was dilated with 1% tropicamide and subretinal injection performed under a dissecting microscope with a pump microinjection apparatus (Harvard Apparatus, Cambridge, MA) and a glass micropipette. Injections were calibrated to deliver two microliters of solution with 50 μg/mL for each compound. A successful injection can be measured by creation of a small subretinal bleb. The number of animals in each group was 8 to 12 to ensure the statistical power.

### Immunofluorescence staining

Whole eyes from the above-mentioned mice were perfusion fixed with 4% paraformaldehyde (PFA) and extracted for cryosection into 10 μm sections. Sections were stained with Alexa Fluor 488 conjugated isolectin (GS-IB 4) (Catalog # I21411, ThermoFisher Scientific) for neovascularization and primary antibodies against F4/80 (Invitrogen) for macrophage infiltration, as well as other proteins of interest including HTRA1, VEGF, phosphor-β-catenin (Cell Signaling Tech), which were detected with a fluorescent-conjugated secondary antibody of different wavelength to examine the location and quantity of these proteins. Nuclei were stained with 4',6-diamidino-2-phenylindole (DAPI). We visualized stain co-localization via the surface plot for pixel density generated by ImageJ.

### Laser-induced CNV, intravitreal injection, and quantification of CNV lesions

C57Bl/6 mice of 6 to 8 weeks were anesthetized by intraperitoneal injection of 100 mg/kg ketamine and 10 mg/kg xylazine, and tropical anesthesia was achieved by 0.1% proparacaine (Allergan Inc., Irvine, CA) eyedrops. After pupils were dilated with topical 0.5% tropicamide (Alcon), laser photocoagulation (532 nm, 75μm spot, 100 mW, 100ms) was performed through a slit-lamp delivery system of an OcuLight GL Photocoagulator (Iridex, Mountain View, CA) bilaterally in each mouse. Four spots were placed at approximately 3, 6, 9, and 12-o’clock and 2 optic disk diameters around the optic disk. All burns had the appearance of a bubble, and those with subretinal bleeding were excluded in this study. Following laser application, intravitreal injections of 2 μL different compound were performed immediately by group. The final concentration of compound in vitreous approximately for Na-LDL and OxLDL was 50 μg/ml; HTRA1 was 100 ng/ml; and anti-HTRA1 Ab was 1 μg/ml.

Seven days after laser photocoagulation, mice were sacrificed and the eyes were processed for FITC-isolectin stain of CNV lesions according to previous reports.[34] Briefly, after fixation with 4% paraformaldehyde for 2 hours and washing with solution containing 0.2% Triton x-100 in PBS, RPE-choroid-sclera complex was flat-mounted with the RPE side upward by 6 radial cuts, and then incubated with 1:100 Isolectin IB4 Conjugates (Invitrogen) overnight at 4°C. Washed the choroid with PBS for 3 times, then mounted and sealed with the RPE-side facing up. Images of were taken and the area of CNV lesion in each sample was measured using NIH Image J software.

## Results

### Subretinal injection of oxLDL induced vascular growth and macrophage infiltration

**Fig 1:**
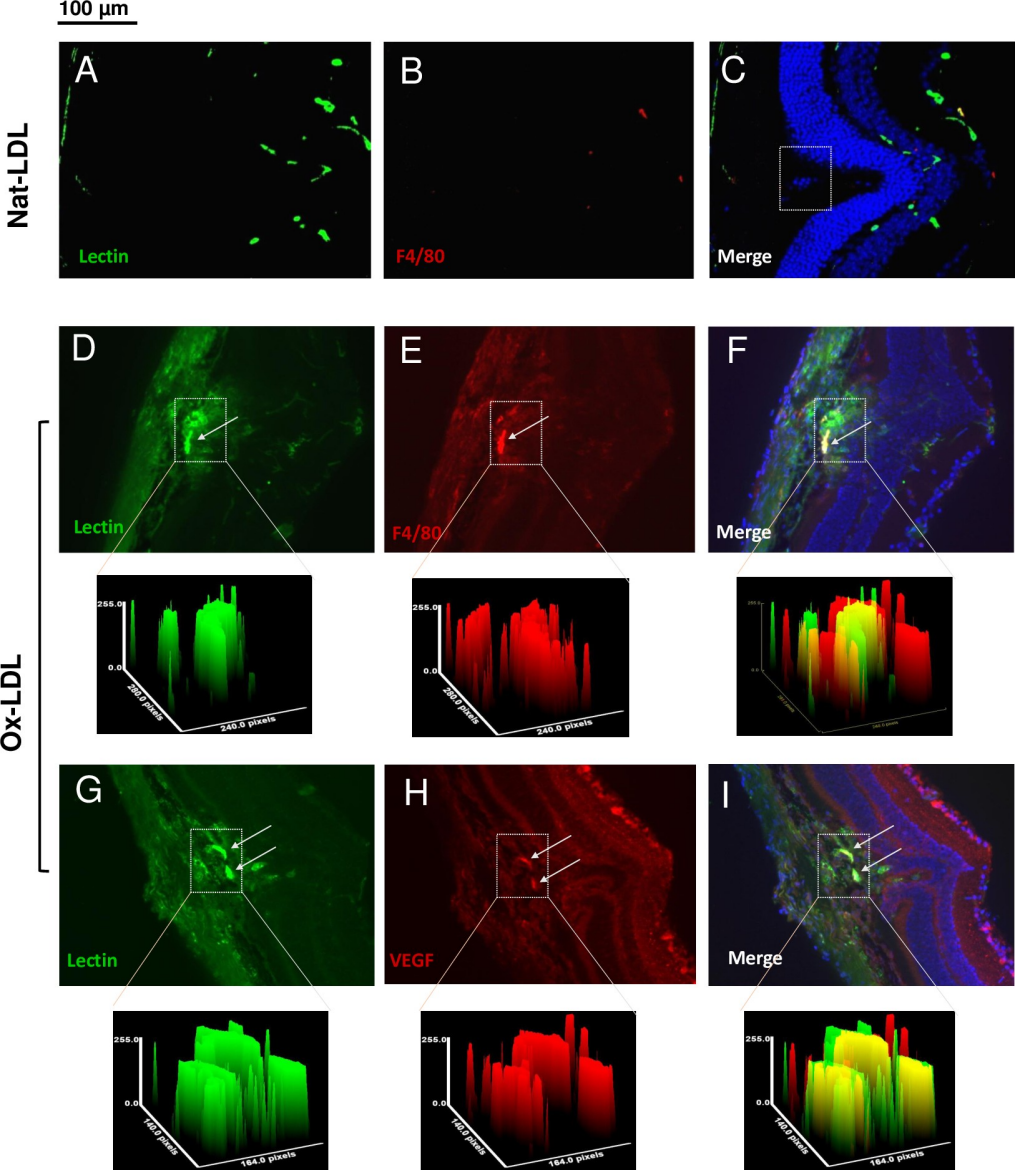
OxPLs stimulate macrophage infiltration, VEGF secretion and neovascularization in a murine model by subretinal injection. Cross-sections of subretinal injection of Nat-LDL (A-C) or oxLDL (D-I) were immunostained with isolectin (green) also to show vascular activity in the subretinal spaces (indicated by an inserted rectangle). Injection with oxLDL resulted in increased vascular activity, which was closely associated with infiltrated macrophages visualized with F4/80 staining (red) (D-F, the inserted area was quantified by pixel density and shown below each panel). Double labeling of neovascularization by isolectin (green), and VEGF (red) (G-I) demonstrates that VEGF was colocalized within a neovascular complex. Cell nuclei were counterstained with DAPI. (Scale bars: 100 μm).

To investigate whether elevated levels of oxLDL in subretinal space can cause lesion-like features resembling CNV, we performed 1.0 μl subretinal injection of 50 μg/ml oxLDL. Two weeks after injection, the animals were sacrificed with CO_2_. The eyes were extracted and fixed with 4% PFA. To access the extent of retinal neovascularization, we stained retinal cross-sections with isolectin (green). Furthermore, monoclonal antibodies were used to target specific markers of macrophage infiltration (F4/80) and VEGF (anti-VEGF). Compared to incubation with Nat-LDL (Fig 1A–1C), oxLDL stimulation was observed to enhance both neovascularization and macrophage infiltration in RPE (Fig 1D–1F, indicated by arrows). In addition, the expression of VEGF was detected at the injection site and it co-localized with neovascularization (Fig 1G–1I, indicated by arrows). The nuclei were counterstained with DAPI.

### OxLDL induces chemoattractant proteins, inflammatory cytokines, HTRA1, and VEGF in ARPE19 cells with temporal fashion

**Fig 2:**
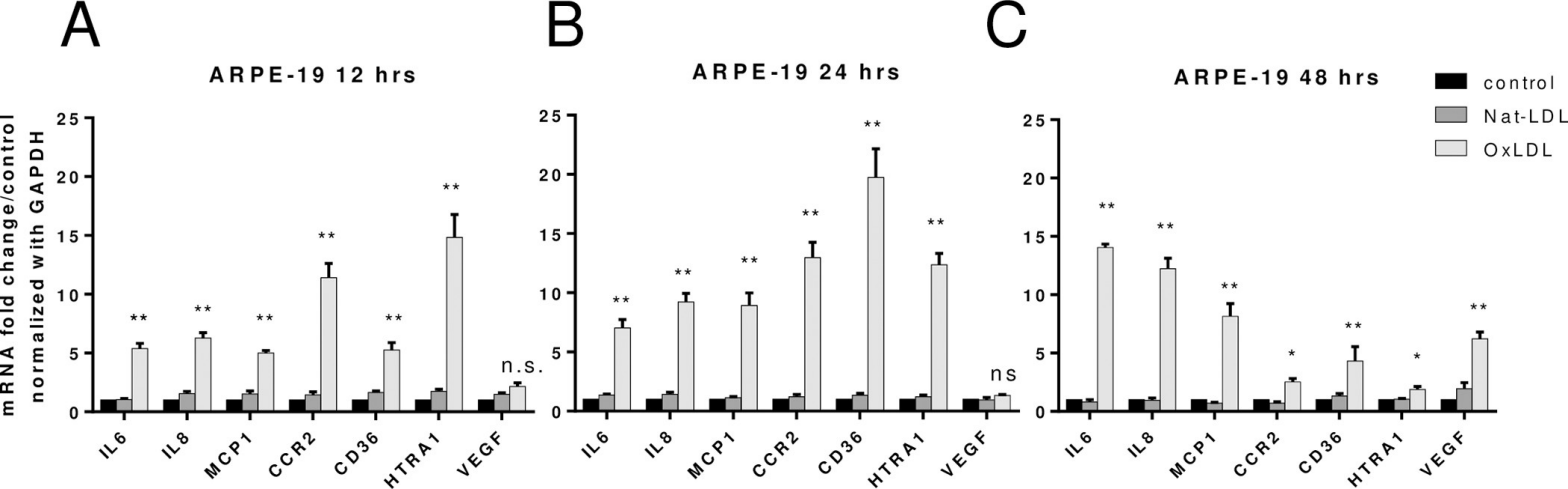
OxPLs stimulate the expression of inflammatory cytokines and chemoattractant proteins in cultured ARPE19 cells. Gene expression as assayed by quantitative PCR on RNA from ARPE19 treated with 50 μg/mL of native-LDL or oxLDL for 12 (A), 24 (B) and 48 (C) hours. Relative mRNA levels of indicated genes (x axis) were calculated by normalizing results with GAPDH and are expressed relative to untreated samples. n = 4. Data are shown as mean ± SEM. *P < 0.05; **P < 0.01.

We used ARPE19 cells to confirm that oxLDL is inflammatory at cellular level and enhances the expression of chemoattractant factors for recruiting circulating monocytes. We treated cultured ARPE-19 cells with 50 μg/mL of native LDL, or oxLDL for 12, 24 or 48 hours. RNA was extracted from the cells and reverse transcribed to cDNA, which was used as a template for gene expression assay by quantitative PCR. Relative mRNA levels for genes of interest were normalized to GAPDH expression level and are expressed as relative-fold-change in comparison to untreated samples. We found that both 12 and 24 hours period after stimulation, oxLDL enhanced the expression of inflammatory cytokines IL6 and IL8, chemoattractant protein MCP-1, the MCP-1 receptor CCR2, and scavenger receptor CD36 (Fig 2). In addition, oxLDL stimulated expression of HTRA1, which is a known genetic factor associated with AMD. Interestingly, though oxLDL incubation did not induce significant expression of VEGF in cells at early stage of stimulation (Fig 2A and 2B), following 48 hours incubation, VEGF expression increased while the expression of CCR2, CD36 and HTRA1 decreased. (Fig 2C)

### In vivo stimulation of oxLDL and HTRA1 elevates the VEGF expression in the eye

**Fig 3:**
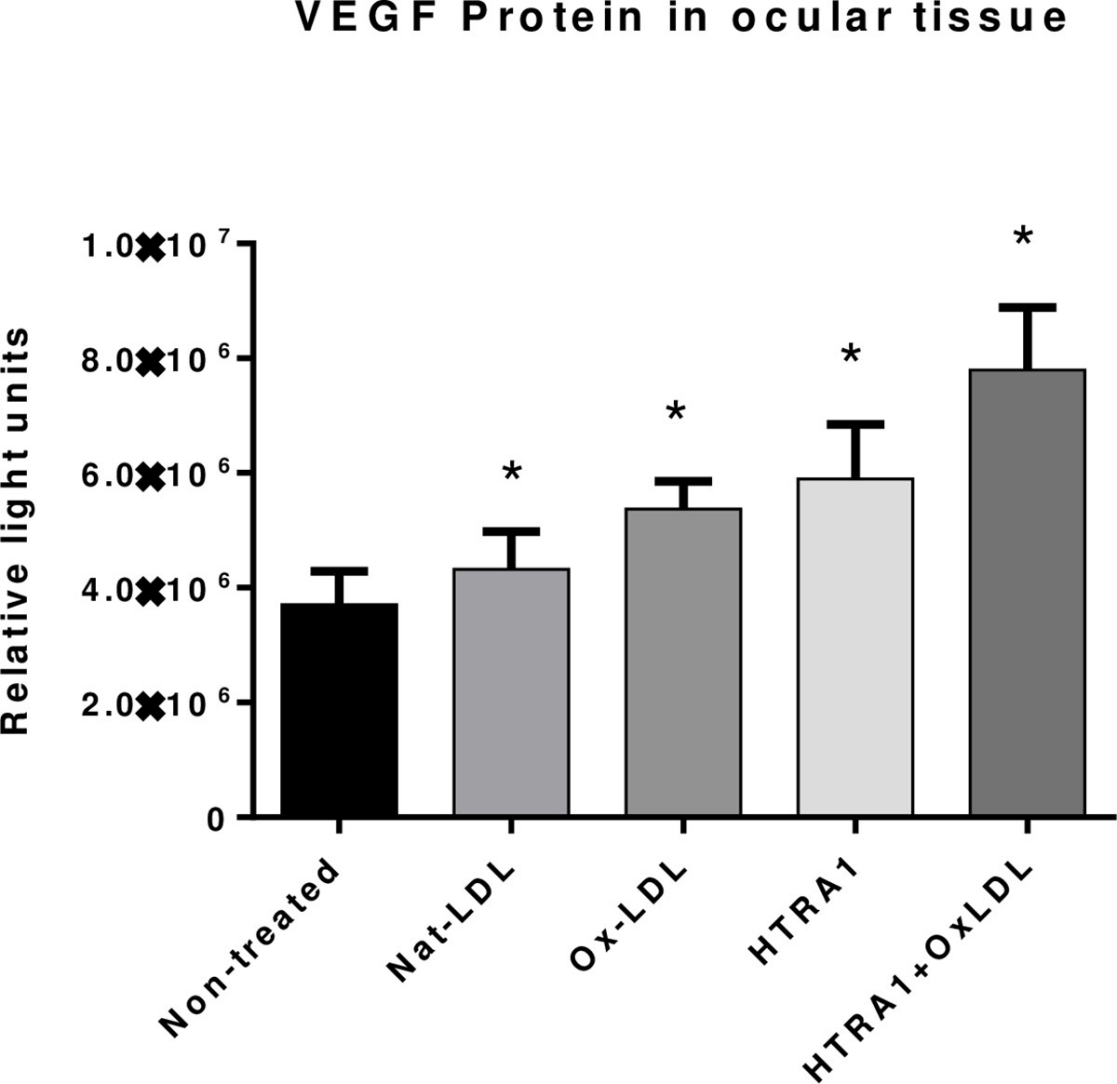
OxPLs and HTRA1 enhanced VEGF expression in the ocular tissue in vivo detected by ELISA. Following intravitreal injection of 50 μg/mL of native-LDL, oxLDL or oxLDL with 100 ng/ml of wild type HTRA1 protein for 7 days, the eyes of the animals were obtained to prepare lysate with RIPA buffer. VEGF protein level in the ocular tissue was assayed by sandwich ELISA with anti-VEGF antibodies. Relative VEGF protein levels are expressed as relative light unit (RLU) for each sample set. n = 4. Data are shown as mean ± SEM. *P < 0.05; **P < 0.01.

We tested overall level of VEGF in the ocular tissue (including retina, RPE and choroid) following the stimulation of oxLDL and/or HTRA1. We grouped Five-week old C57/BL6 mice into four cohorts (N = 4 in each group) and injected intravitreally with oxLDL, or oxLDL + HTRA1 respectively and with Nat-LDL as control and non-treated mice as background control. Seven days post-injection, the animals were sacrificed, and the eyes were collected to prepare lysate for ELISA. The ELISA results showed that the VEGF protein level in the eye was significantly higher in oxLDL + HTRA1 group than in oxLDL or HTRA1 alone groups than in Nat-LDL group (Fig 3).

### HTRA1 synergizes with oxidized phospholipids in activation of inflammatory factors but not VEGF in ARPE-19 cells

**Fig 4:**
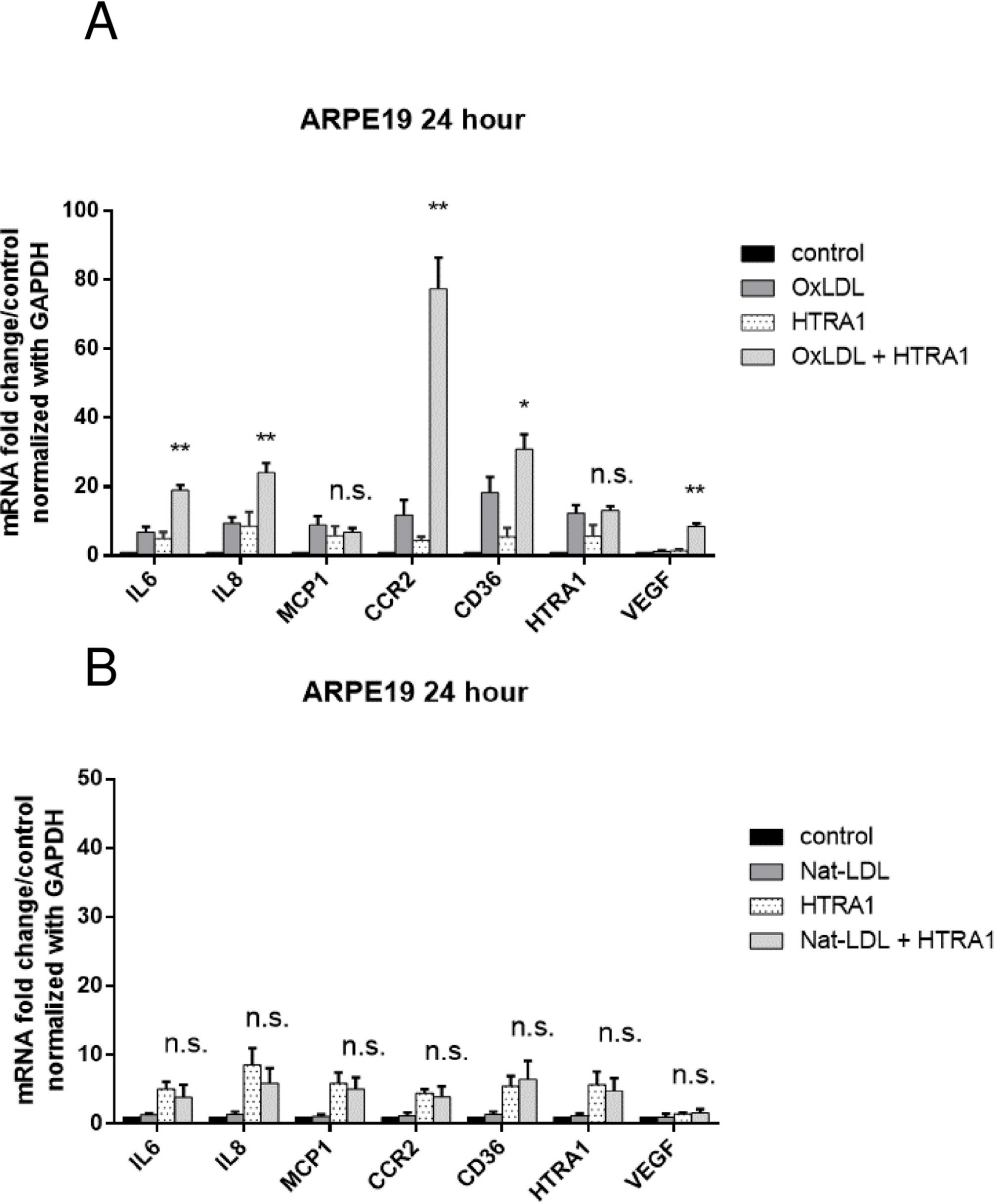
OxPLs and HTRA1 synergistically enhanced VEGF expression. ARPE19 cells were treated with 50 μg/mL of Nat-LDL, or 100 ng/ml of wild type HTRA1 protein, or both Nat-LDL and HTRA1 for 24 hours (A). In parallel, ARPE19 cells were treated with 50 μg/mL of oxLDL, or 100 ng/ml of wild type HTRA1 protein, or both oxLDL and HTRA1 for 24 hours (B). RNA was extracted for gene expression assay by quantitative PCR. Relative mRNA levels of indicated genes (x axis) were calculated by normalizing results with GAPDH and are expressed relative to untreated samples. n = 4. Data are shown as mean ± SEM. *P < 0.05; **P < 0.01.

Given that the significant expression of HTRA1 during the early oxLDL stimulation (12 hours), we investigated whether oxLDL and HTRA1 could cooperate to initiate the inflammatory cascade and trigger subsequent events leading to CNV. Thus, we assayed gene expression by qPCR from ARPE-19 cells treated with 50 μg/mL of oxLDL in either presence or absence of 100 ng/ml of HTRA1 for 24 hours. Our results showed that mRNA levels of inflammatory cytokines IL-6 and IL-8 in ARPE-19 cells were dramatically increased following incubation with HTRA1+oxLDL in comparison to incubation with oxLDL alone (Fig 4A). However, incubation of ARPE-19 cells with HTRA1+Nat-LDL there was no significant increase in mRNA levels of IL-6 and IL-8 compared to incubation with HTRA1 alone (Fig 4B). These data demonstrated the synergetic effect of HTRA1 and oxLDL in activation of inflammation. We have also observed significant increase of CCR2 and CD36 expression, as well as the increase of VEGF expression when incubated with HTRA1+oxLDL.

### Wnt signaling is necessary for VEGF expression and is mediated with resident macrophages

**Fig 5:**
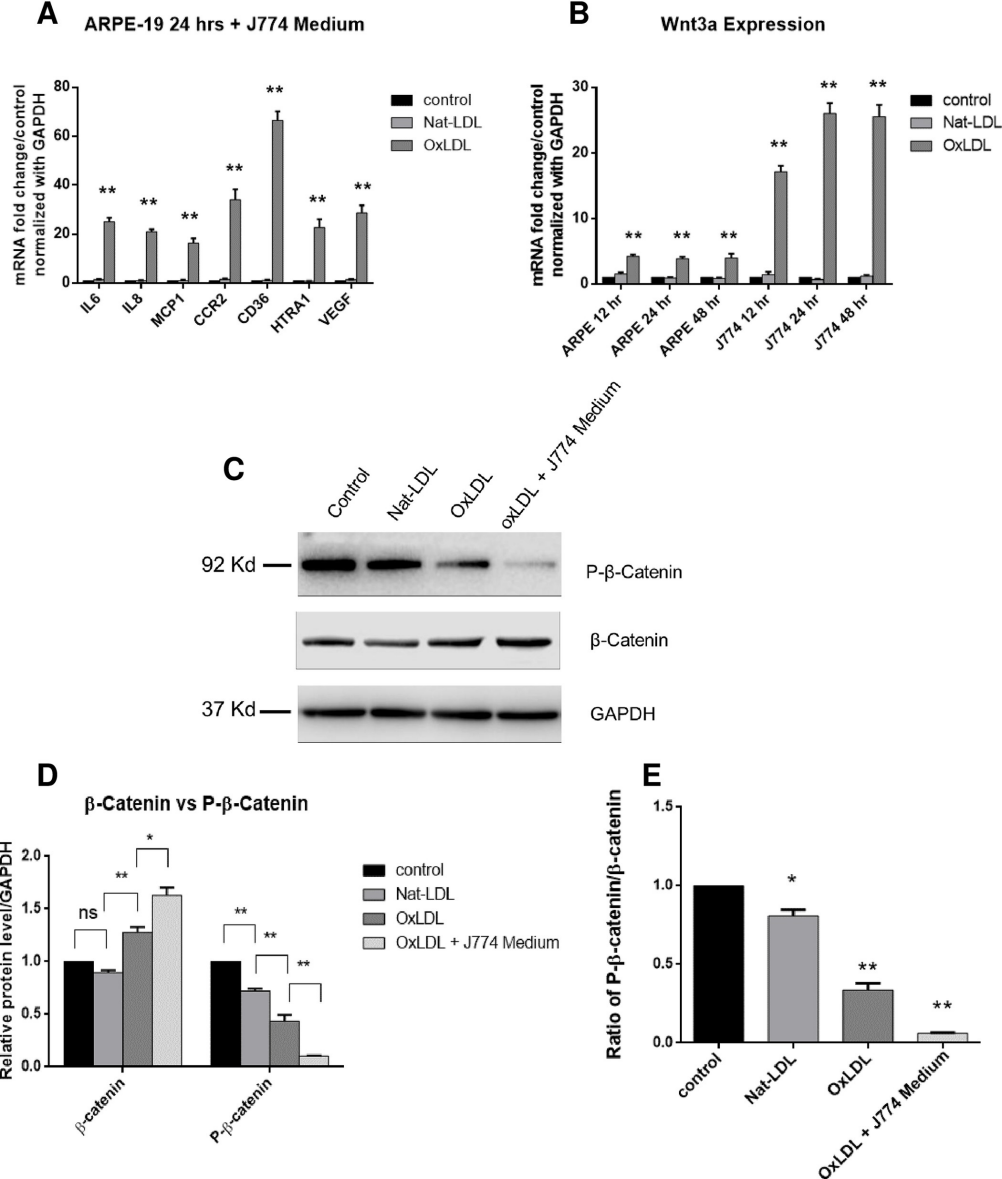
The macrophage with oxLDL conditioned medium enhanced the expression of inflammatory cytokines and chemoattractant proteins, as well as VEGF in cultured ARPE19 cells. **(A).** OxPLs stimulate the expression of Wnt3A in J774 macrophages **(B).** Relative mRNA levels were calculated by normalizing results with GAPDH and are expressed relative to untreated samples. n = 4. Data are shown as mean ± SEM. *P < 0.05; **P < 0.01. Western blot analysis for β-catenin and phospho-β-catenin proteins in ARPE19 cells following the treatment of Nat-LDL, oxLDL or oxLDL with J774 conditioned medium. **(C)** Representative Western blots image. **(D)** Relative levels of β-catenin and phospho-β-catenin proteins normalized to GAPDH. **(E)** The ratio of phospho-beta-Catenin to total beta-Catenin protein. N = 3; means ± standard deviations are shown. P values tested against control are indicated. n.s., non-significant.

In order to study the molecular basis of why VEGF expression was induced by oxLDL *in vivo* but not in cell culture in early stage, we hypothesized that oxidative stress induces macrophages to secrete molecular signal(s) that trigger an increase in VEGF expression. To test this hypothesis, we stimulated J774 cells with oxLDL for 24 hours, and added the conditioned supernatant to ARPE19 cell culture for another 24 hours incubation. We found that the addition of conditioned supernatant from J774 to ARPE-19 cell culture enhanced the expression of not only inflammatory cytokines and chemoattractant proteins, but also VEGF (Fig 5A). Since macrophages mediate much inflammatory response and are associated with drusen formation and CNV, we examined inflammatory gene expression in mouse macrophage J774 stimulated with oxLDL and Nat-LDL. We found that oxLDL was able to induce much higher expression of Wnt3A in J774 macrophages than in ARPE19 cells (Fig 5B). Wnt3A is an important Wnt protein that induces cellular proliferation by both canonical and non-canonical Wnt/β-catenin pathways. The presence of Wnt prevents phosphorylation and degradation of β-catenin, which translocates into the nucleus and activates transcription of target genes including VEGF-A. [35, 36] We also examined the Wnt signaling pathway effector β-catenin and phosphorylated β-catenin (P-β-catenin) of ARPE19 cells upon stimulation of oxLDL in the presence or absence of J774 macrophage conditioned medium. We found that P-β-catenin was only slightly reduced when ARPE-19 cells were incubated with oxLDL in basal media, but P-β-catenin decreased significantly when conditioned medium was added to ARPE-19 cell culture (Fig 5C and 5D).

### Antibody that inhibits HTRA1 activity can alleviate its potential to stimulate inflammatory and angiogenic factors

**Fig 6:**
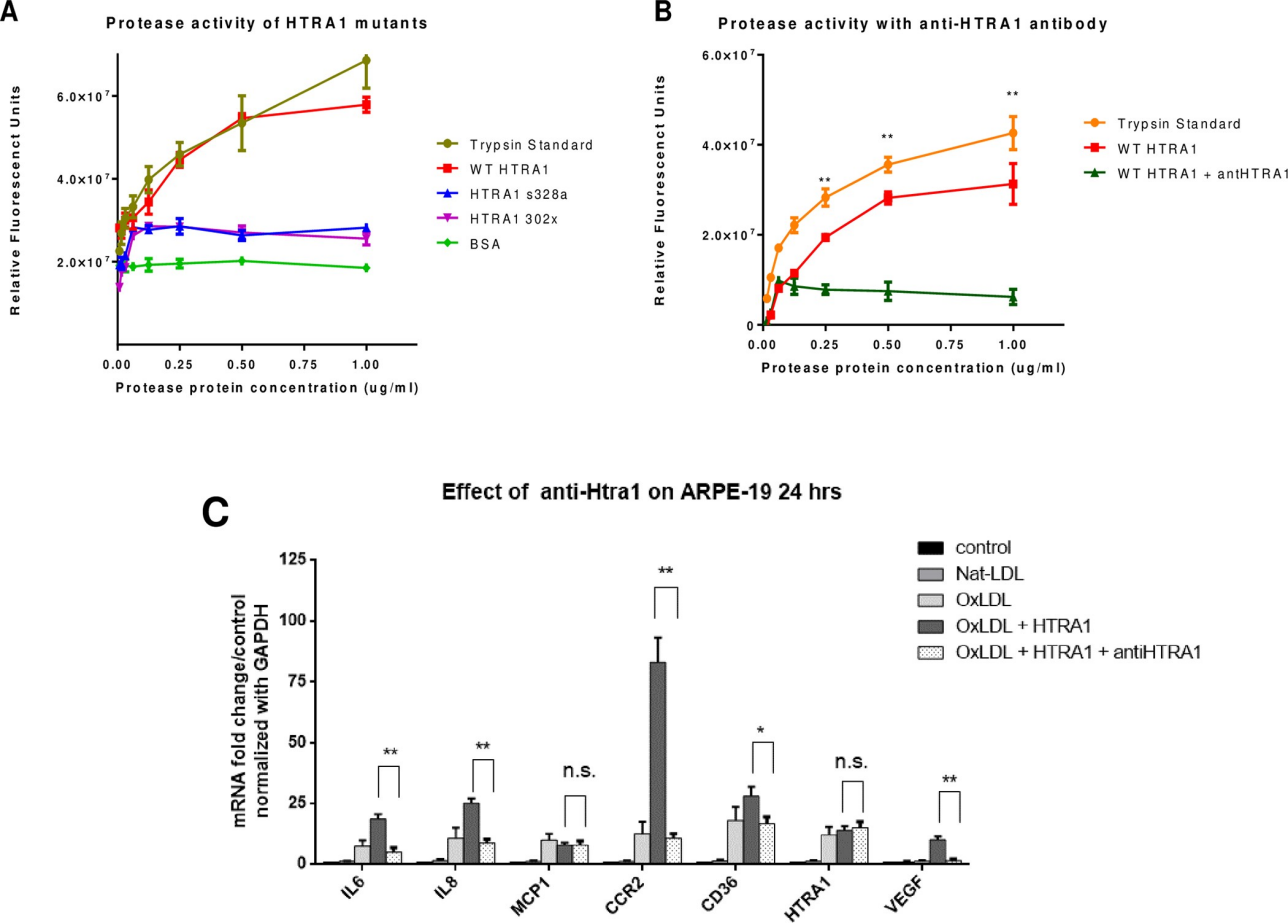
Anti-HTRA1 antibody fragment inhibits HTRA1 protease activity and neutralizes HTRA1 induced inflammation. (A) Wild type HTRA1 and its mutants, s328a lacking the protease activity and 302x removing PDZ domain were assayed for their protease activity using the Pierce Fluorescent Protease Assay. Trypsin is included in the kit was used as positive control. (B) Increasing concentration of wild type HTRA1 alone or with fixed amount of anti-HTRA1 prep (2.0 μg/mL) were assayed for protease activity. (C) Gene expression as assayed by quantitative PCR on mRNA from ARPE-19 cells treated with 100 ng/ml of HTRA1 alone or HTRA1 + 1.0 μg/mL of scFv anti HTRA1 fragment for 18 hours. Relative mRNA levels of indicated genes were calculated by normalizing results with GAPDH and are expressed relative to untreated samples. Data are shown as mean ± SEM. N = 4, *P < 0.05; ** <0.01.

To further confirm that HTRA1 activity is the important factor that resulted in enhanced expression of inflammatory cytokines and angiogenic factor, we performed protease activity inhibition experiment with an antibody that can neutralize HTRA1’s enzymatic activity. We first assayed the protease activity of wild-type HTRA1 and its variants S328A and 302X using the Pierce Fluorescent Protease Assay. The S328A variant lacks protease activity, whereas the 302X variant lacks the PDZ domain. Fig 6A shows the relative protease activity of wild type HTRA1, variant s328a, and variant 302x compared to trypsin-standard as positive control. Fig 6B shows the relative protease activity of wild-type HTRA1 in either presence or absence of fixed amount of anti-HTRA1 antibody in comparison with trypsin-standard. This figure demonstrates that anti-HTRA1 successfully neutralizes the protease activity of HTRA1. Next, we performed the gene expression assay by qPCR on ARPE19 cells treated with 100 ng/ml either HTRA1 alone or HTRA1 + 1.0 μg/mL of anti HTRA1 antibody for 18 hours. Fig 6C demonstrates that anti-HTRA1 successfully neutralizes the effect of HTRA1 on the enhancement of inflammatory cytokines and VEGF gene expression.

### Laser-induced CNV sizes are enlarged with the presence of oxLDL and HTRA1, but neutralized with anti-HTRA1

**Fig 7:**
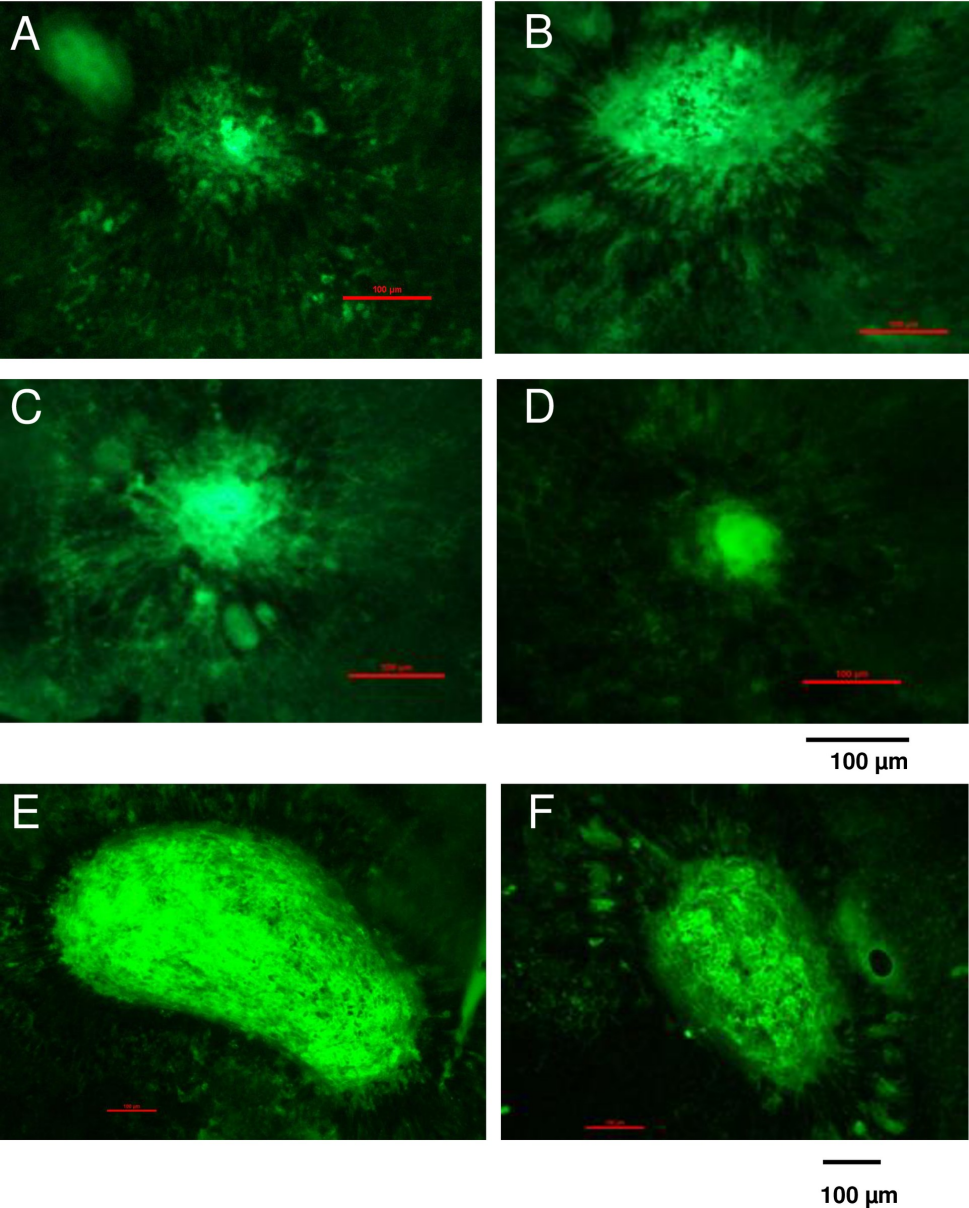
OxPLs and HTRA1 affect neovascularization in a murine model of laser induced CNV. Seven days after laser treatment, mice were sacrificed and choroidal flat mounts were generated. AlexaFluor-conjugated isolectin was used for CNV immunolabeling. Representative images of CNV lesions in choroidal flat mounts from laser photo-coagulant treated eyes with injection of Nat-LDL (A), oxLDL (B), HTRA1 (C), HTRA1 + anti HTRA1 (D), oxLDL + HTRA1 (E), or oxLDL + HTRA1 + ant-HTRA1 (F). (Scale bars: 100 μm).

**Fig 8:**
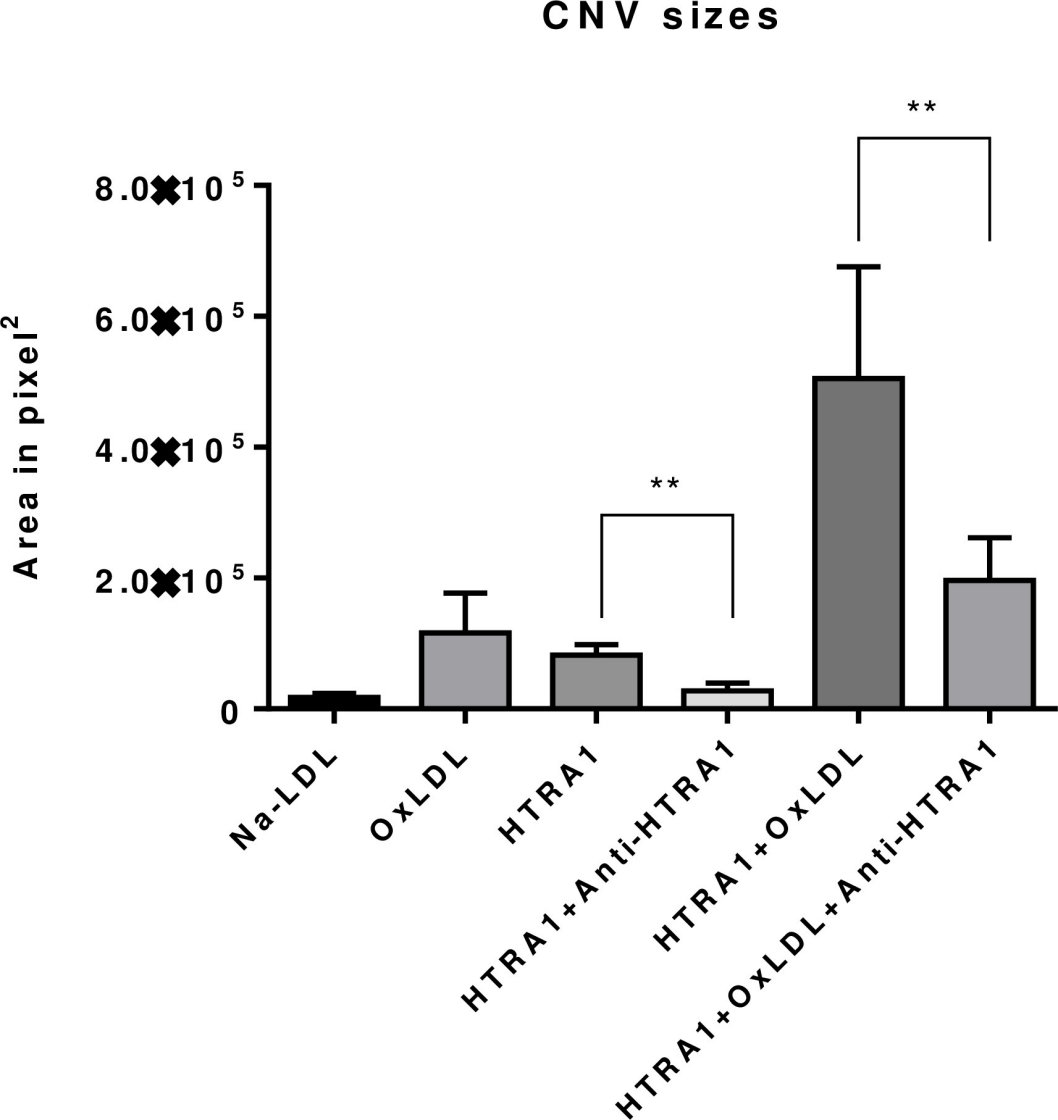
The CNV lesion size was measured using Velocity Software (PerkinElmer, Waltham, MA) and expressed as mm^2^ (n = 4, means ± SEM; *P < 0.01 vs. WT).

Finally, we performed laser-induced CNV as previously described.[34] 6–8 week old C57BL/6 mice were separated into 3 cohorts with 4 mice in each group (Table 2). Following laser photocoagulation treatment, the animals immediately received intravitreal injection of indicated compound. Seven days after laser photocoagulation, the mice were sacrificed, and the eyes were processed for flat mount and FITC-isolectin stain to visualize CNV lesions. Representative images are shown in Fig 7. (A-F: Notice that the scale bar in images E and F is doubled due to the larger lesion sizes). The results in Fig 8 show that the average lesion sizes in the presence of either oxLDL or HTRA1 were increased significantly compared to Nat-LDL. With the presence of both oxLDL and HTRA1, the laser-CNV lesions were increased significantly. The data also demonstrate that the anti-HTRA1 antibody was able to neutralize the effect of HTRA1 on lesion size.

**Table 2:** Grouping of experimental animals.

Cohort Groups (n = 4)	Right (OD)	Left (OS)
**1**	Na-LDL (2 μL of 100 μg/ml)	OxLDL (2 μL of 100 μg/ml)
**2**	HTRA1, (2 μL of 200 ng/ml)	HTRA1 (1 μL of 400 ng/ml) Anti-HTRA1 Ab (1 μL of 4 μg/ml)
**3**	HTRA1 (1 μL of 400 ng/ml) Ox-LDL (1 μL of 200 μg/ml)	HTRA1 /Ox-LDL/ Htra-1Ab (2 μL of mix containing 100 μg/ml oxLDL; 200 μg/ml Ntra1 and 2 μg/ml anti-HTRA1)

## Discussion

Genome-Wide Association Studies (GWAS) have catalyzed significant progress towards elucidating the molecular basis of complex trait diseases.[37] However, a substantial gap remains between the association of a trait with genetic variants and understanding disease mechanisms. As a complex retinal degenerative disease, AMD illustrates this challenge. Even though the pathology and prevalence of this disease has been studied for more than a century, there remain substantial questions regarding the underlying biochemical and cellular events that give rise to this disease. Our study has aimed to close this gap by investigating a novel mechanistic interplay among oxidative stress, variants of the HTRA1 gene, and expression levels of VEGF.

Our investigation was motivated by the differential inflammatory response to oxidative stress between *in vitro* ARPE-19 culture and *in vivo* C57BL/6 murine model. When ARPE-19 cells were incubated with only oxLDL, statistically significant (p < 0.01) increases in the expression of IL-6, IL-8, MCP-1, CCR2, CD36, and HTRA1 were observed 24 hours after the addition of oxLDL (see Fig 2). Interestingly, the expression of VEGF did not significantly increase until 48 hours after the addition of oxLDL, at which time the expression of CCR2, CD36, and HTRA1 had begun decreasing to pre-oxLDL addition levels (see Fig 2). In isolation, these data provide *prima facie* evidence for a time-dependent retinal response to oxidative stress, whereby there is a time delay between the recruitment and activation of inflammatory factors and the start of angiogenesis. However, we explored the possibility that this time-delay in VEGF upregulation is an artifact of the ARPE-19 culture system rather than an intrinsic feature of the retinal response to oxidative stress.

Unlike the ARPE-19 culture system, retinas are composed of many different cell types, some permanent and some transient, which may play specific roles in AMD pathogenesis. Immunohistochemistry of C57BL/6 murine retinas following subretinal injection of oxLDL provided qualitative evidence for the co-localization of VEGF and macrophages within the retina (see Fig 1). Furthermore, our in vivo data by introducing those stimulants (e.g. oxLDL, HTRA1 or both) through intravitreal injection demonstrated that oxidized PLs stimulated the higher VEGF protein levels in the eye. In the presence of oxPLs, with HTRA1 level elevated, the VEGF expression in the ocular tissues was further enhanced (Fig 3).

Based on this preliminary evidence, we hypothesized that infiltrating macrophages may secrete a signal that triggers upregulation of VEGF expression 24 hours or less after addition of oxLDL. In order to test this hypothesis, we cultured J774 macrophages with oxLDL for 24 hours and transferred the conditioned medium to the ARPE-19 culture. Following oxLDL incubation of the ARPE-19 culture containing the conditioned medium for another 24 hours, gene expression was assessed via qPCR. Unlike the initial ARPE-19 culture system, the ARPE-19 culture system containing J774 conditioned media *did* exhibit statistically significant (p < 0.01) upregulation in VEGF expression within 24 hours after addition of oxLDL (see Fig 5A). Furthermore, the expression of VEGF in the ARPE-19 culture with conditioned media after 24 hours oxLDL incubation was 2-fold greater than the expression of VEGF in the ARPE-19 culture with basal media after 48 hours oxLDL incubation (compare Figs 2C and 5A). This result provided the first evidence that macrophage infiltration into the neural retina may be *necessary* for sufficient and timely upregulation of VEGF that is essential to the development of choroidal neovascularization.

The results of the preceding experiment confirmed that upregulation of VEGF could be expedited and amplified by the addition of J774 conditioned medium. However, the factors in the J774 conditioned media that were responsible for the upregulation in VEGF expression remained unknown. Under oxidative stress, it is known that resident macrophages express numerous proteins that activate the Wnt signaling pathway.[38, 39] One of these proteins, Wnt3A, binds to the Fz/LRP6 coreceptor complex, preventing the phosphorylation and subsequent ubiquitin-mediated degradation of β-catenin.[40] Accumulation of cytosolic β-catenin results in its translocation to the nucleus, where it interacts with lymphocyte-enhancing factor (LEF)/T-cell factor (TCF) in order to activate the transcription of numerous target genes, including VEGF-A.[36, 41] This proposed link between macrophage infiltration and VEGF upregulation is supported by the data presented in this report.

We found that when cultured macrophages were treated with oxLDL, Wnt3A expression in these cells significantly increased (p<0.01) (see Fig 5B). When the conditioned medium was added to the cultures of APRE19 cells with oxLDL, the ARPE-19 cells responded not only with increased expression of IL6 and IL8, but also increased expression of VEGF (see Fig 5A). In addition, at the protein level, we found that the phosphorylation of β-catenin, the effector of Wnt signaling pathway, was significantly decreased when J774 macrophages conditioned medium was added. First, Western blot analysis of the J774 conditioned medium treated with oxLDL exhibited statistically significant (p < 0.05) increase in unphosphorylated β-catenin and statistically significant (p < 0.01) decrease in phosphorylated P-β-catenin (see Fig 5C and 5D). Second, qPCR analysis of the J774 conditioned medium 24 hours after addition of oxLDL indicates statistically significant upregulation in Wnt3A compared to base ARPE-19 medium 24 hours after addition of oxLDL (4.03±0.411 vs 24.9±2.36, p < 0.01, APRE-19 24 hours vs J774 24 hours, two-tail unpaired t-test) (see Fig 5B). These two lines of evidence suggest an important role for the Wnt pathway in initiating the upregulation in VEGF expression that is essential for the development of choroidal neovascularization in wet AMD.

Lastly, our study sought to elucidate the role of HTRA1 in modulating the development of choroidal neovascularization *in vitro* and *in vivo*. Our results demonstrate that HTRA1 synergizes with oxLDL by increasing the inflammatory response to oxidative stress. After 24 hours of incubation with oxLDL and HTRA1, ARPE-19 cells in basal media exhibited statistically significant increases (p < 0.01) in IL-6, IL-8, CCR2, and VEGF compared to incubation with oxLDL alone (see Fig 4A). We verified that the effect of HTRA1+oxLDL on gene expression is synergistic and not merely additive (see Fig 4A). These increases in gene expression, particularly the VEGF expression, were reversed by the inclusion of anti-HTRA1 monoclonal antibody (mAb) in the culture media, demonstrating the efficacy of our antibody at neutralize the inflammatory effect of HTRA1 on cultured ARPE-19 (see Fig 6C). The synergistic inflammatory effect of HTRA1 and the neutralizing capability of our anti-HTRA1 mAb were successfully reproduced *in vivo* (Fig 7). C57BL/6 mice that were subjected to laser-induced CNV exhibited statistically significant (p < 0.01) increases in CNV lesion size following subretinal injection of oxLDL and HTRA1 compared to either oxLDL or HTRA1 alone. Furthermore, these mice exhibited statistically significant (p < 0.01) decreases in CNV lesion size in the presence of anti-HTRA1 mAb, thereby demonstrating the efficacy of our antibody *in vivo*.

These synergistic inflammatory effect of HTRA1 and oxLDL as well as the efficacy of anti-HTRA1 mAb convincingly demonstrate that HTRA1 influences the inflammatory response and choroidal neovascularization resulting from oxidative stress. However, the underlying mechanism for this affect remains less clear. HTRA1 is a serine protease, with variants in either the protein or its promoter being consistently linked to AMD pathogenesis and progression. One mode by which HTRA1 may modulate AMD pathogenesis is by compromising the integrity of the Bruch’s membrane,[26] which may lead to increased diffusivity of inflammatory or angiogenic factors across Bruch’s membrane.[40] In a normal eye, the choroidal vasculature is well-defined and is separated from the neural retina via Bruch’s membrane and the RPE. In an eye with wet AMD, the integrity of the vascular compartmentalization is compromised, possibly due to degradation of ECM proteins that comprise the Bruch’s membrane and tight junctions of the RPE. As angiogenic signals (e.g. VEGF) pass from the choriocapillaris into the neural retina, choroidal neovascularization and other clinical hallmarks of wet AMD result.

However, the results of this study suggest an additional mechanism by which HTRA1 may affect AMD pathogenesis. HTRA1 is known to target many different proteins for degradation, including the components of extracellular matrix (ECM) [42] that regulate the function of canonical Wnt signaling pathway.[43] In addition, Dickkopf-related protein 1 (DKK-1) is an inhibitor of the Wnt pathway [44, 45] that disrupts the Fz/LRP6 coreceptor complex,[40, 46] thereby preventing the downstream Wnt signaling cascade. Mutations in the promoter or the coding sequence for HTRA1 that result in either overexpression or increased proteolytic activity would be expected to result in decreased levels of DKK-1. As DKK-1 levels decrease, the Wnt signaling cascade would be less inhibited, resulting in increased cytosolic concentration of β-catenin. Higher concentration of cytosolic β-catenin would result in increased nuclear translocalization and, therefore, increased expression of VEGF. While the results of this study suggest that HTRA1 affects VEGF expression by two orthogonal mechanisms, additional experimentation would be necessary in order to determine the relative contribution of each of these mechanisms to the development of choroidal neovascularization.

Regardless of the mechanism by which HTRA1 affects the retinal response to oxidative stress, our study demonstrates has a significant and reproducible effect on local inflammation and choroidal neovascularization. Hence, we propose HTRA1 as a novel therapeutic target for patients with wet AMD. Currently, the primary treatment for wet AMD is anti-VEGF mAb therapeutics.[17] Similarly, an anti-HTRA1 therapeutic would result in a decrease in VEGF expression, thereby slowing or halting choroidal neovascularization. Furthermore, unlike anti-VEGF treatment, anti-HTRA1 treatment may permit endogenous restoration of Bruch’s membrane integrity. If the proteolytic activity of HTRA1 were neutralized via anti-HTRA1 therapeutic, the retina may be able to synthesize elastin, vitronectin, and other essential components of the Bruch’s membrane, thereby restoring its essential transport regulatory functions. If this could be demonstrated experimentally, then anti-HTRA1 may be an addition or complement to the existing treatment.

## Conclusions

This paper investigated the relationship between oxidative stress and a common polymorphism in the HTRA1/ARMS2 region on chromosome 10q26 on AMD pathogenesis. *In vivo*, we found that subretinal injection of oxLDL resulted in macrophage infiltration, increased VEGF expression, and neovascularization. However, *in vitro* incubation of ARPE-19 cells with oxLDL induced expression of inflammatory cytokines and chemoattractant proteins but failed to increase VEGF expression. We revealed that oxLDL stimulates macrophages to express Wnt3a, which is essential to trigger a strong and evaluated expression of vascular factors through the Wnt signaling pathway that are necessary for CNV in RPE. Furthermore, we demonstrated that oxLDL not only induces the expression of inflammatory cytokines and chemoattractant proteins required for macrophage infiltration, but also the expression of HTRA1, which synergizes with oxLDL in enhancing the inflammatory response. Hence, we investigated HTRA1 as a potential therapeutic target in order to prevent excessive VEGF expression in patients with AMD. We performed *in vitro* assessment confirming the neutralizing effect of anti-HTRA1 antibody on inflammatory and angiogenic responses to oxidative stress. Finally, we used a mouse laser-CNV model of AMD to demonstrate that CNV sizes were enhanced by HTRA1 and/or oxLDL following laser photo-coagulation and that such enhancement can be neutralized with antibody to HTRA1, indicating the therapeutic potential of anti-HTRA1 in treating patients with wet AMD.

## Supporting information

S1 Data (ZIP)
